# Fairness and generalizability of OCT normative databases: a comparative analysis

**DOI:** 10.1186/s40942-023-00459-8

**Published:** 2023-08-21

**Authors:** Luis Filipe Nakayama, Lucas Zago Ribeiro, Juliana Angelica Estevão de Oliveira, João Carlos Ramos Gonçalves de Matos, William Greig Mitchell, Fernando Korn Malerbi, Leo Anthony Celi, Caio Vinicius Saito Regatieri

**Affiliations:** 1 Laboratory of Computational Physiology, Massachusetts Institute of Technology, 77 Massachusetts Ave, Cambridge, MA 02139 United States of America; 2 Department of Ophthalmology, São Paulo Federal University, Sao Paulo, SP Brazil; 3 University of Porto, Porto, Portugal; 4 Department of Ophthalmology, Royal Victorian Eye and Ear Hospital, Melbourne, Australia; 5 Department of Biostatistics, United States of America, Harvard TH Chan School of Public Health, Boston, MA United States of America; 6 Department of Medicine, Beth Israel Deaconess Medical Center, Boston, MA United States of America

**Keywords:** Optical coherence tomography, Fairness, Generalizability, Database, Supervised machine learning

## Abstract

**Purpose:**

In supervised Machine Learning algorithms, labels and reports are important in model development. To provide a normality assessment, the OCT has an in-built normative database that provides a color base scale from the measurement database comparison. This article aims to evaluate and compare normative databases of different OCT machines, analyzing patient demographic, contrast inclusion and exclusion criteria, diversity index, and statistical approach to assess their fairness and generalizability.

**Methods:**

Data were retrieved from Cirrus, Avanti, Spectralis, and Triton’s FDA-approval and equipment manual. The following variables were compared: number of eyes and patients, inclusion and exclusion criteria, statistical approach, sex, race and ethnicity, age, participant country, and diversity index.

**Results:**

Avanti OCT has the largest normative database (640 eyes). In every database, the inclusion and exclusion criteria were similar, including adult patients and excluding pathological eyes. Spectralis has the largest White (79.7%) proportionately representation, Cirrus has the largest Asian (24%), and Triton has the largest Black (22%) patient representation. In all databases, the statistical analysis applied was Regression models. The sex diversity index is similar in all datasets, and comparable to the ten most populous contries. Avanti dataset has the highest diversity index in terms of race, followed by Cirrus, Triton, and Spectralis.

**Conclusion:**

In all analyzed databases, the data framework is static, with limited upgrade options and lacking normative databases for new modules. As a result, caution in OCT normality interpretation is warranted. To address these limitations, there is a need for more diverse, representative, and open-access datasets that take into account patient demographics, especially considering the development of supervised Machine Learning algorithms in healthcare.

## Introduction

In Artificial Intelligence (AI) and Machine Learning (ML), computational models predict results from connections in abstractions of inputted data [1]. In supervised ML, label records are used during model development, but a reliable labeling process can be a laborious and expensive process, often taking advantage of reports in the dataset development process [2].

With the proliferation of digital devices in healthcare, an enormous volume of data is accrual [3]. However, there is a risk of bias when the data, especially that used to determine ‘normal’ parameters, is not representative of the heterogeneous populations for whom the technology is used, with unfair and harmful algorithms outcomes against underrepresented populations [4–6].

Optical Coherence Tomography (OCT) is a biomedical non-invasive imaging exam that generates low-coherence interferometry to generate three-dimensional images of biological tissues [7]. The OCT makes inferences on tissue characteristics by interpreting reflections of a light beam at different tissue depths and has evolved from a time domain to frequency domain technology, which has a more sensitive image capacity, faster-capturing speed, better image depth, and enhanced image availability [7].

The OCT scanner is now widely implemented throughout healthcare, such as ophthalmology, cardiology, dermatology, and dentistry [8–12]. In ophthalmology, the OCT is routinely used to aid the diagnosis of various conditions, including retinal diseases[13], glaucoma [14], and cornea diseases[15]. OCT scans are widely implemented in helping the decision-making for macular diseases, such as anti-vascular treatment for macular edema in central retina vein occlusion [16], age-related macular degeneration [17], and diabetic macular edema [18].

The color-based scale utilized in OCT analysis is established based on the percentiles of normal distributions within the normative database for each A-scan location among same-age individuals. The scale is divided into four color-coded zones: the white area is above 95% of the distribution, the green area is between 5% and 95%, the yellow between 1% and 5%, and the red below 1%. However, the normality report is defined based on the percentage of the thickest or thinner measurements across the color-based scale.

Given the relatively limited demographic representativeness of data used to form ‘normal’ parameters for each machine’s database, interpretation of the degree of disease severity (and how much it differs from ‘normal’ parameters) can be difficult for clinicians and input bias in AI and ML models [19, 20]. Moreover, the lack of normative data for the under-18 population excludes pediatric patients from the analysis [21].

Despite its widespread use in ophthalmic practice, OCT normative databases applied in OCT analysis are often a limited, static, private, and lacking assessment of generalizability and fairness – especially when used for underrepresented populations. Among the different ophthalmological subspecialties, mainly glaucoma and retina, rely on normative data analysis in clinical practice.

This article aims to evaluate and compare normative databases of different OCT machines, analyzing patient demographic, contrast inclusion and exclusion criteria, diversity index, and statistical approach to assess their fairness and generalizability. Our study compared the OCT’s normative databases diversity with China, the United States of America, and Brazil’s demographics.

## Materials and methods

This study was conducted exclusively with publicly available data, in accordance with the Helsinki Declaration. We analyzed the layer measurement data extracted from each OCT segmentation model. These segmentation algorithms have not been evaluated in this study.

### Datasource

For the current study, we analyzed data retrieved from the United States (US) Food and Drug Administration (FDA) medical devices database and the OCT equipment user manual of the following contemporary OCT equipment: Carl Zeiss Cirrus, RTVue Avanti, Heidelberg Spectralis, and Topcon Triton.

### Variables

We extracted variables from the database included: (1) the number of included eyes and patients, (2) inclusion and exclusion criteria, and (3) the statistical approach. The demographic variables we document here are (1) sex, (2) race and ethnicity, (3) participants’ age, and (4) population country. Race and ethnicity variables were interpreted according to the NIH and US Federal Standard [22, 23].

### Diversity assessment

To assess the diversity, we applied the Shannon diversity index (*H*=−∑[(*p*i​)×ln(*p*i​)]) on OCT normative databases participants’ race and sex. We compared the OCT diversity index with the sex and race diversity of the ten most populous countries’ world populations [24]. Additionally, we compared the open-angle glaucoma sex and race diversity indexes from two meta-analyses [25, 26].

## Results

The FDA has two regulatory pathways to approve medical devices in the USA the FDA approval, which is required for new and innovative devices, and the FDA 510(k) clearance when the device is equivalent to an already FDA-approved one on the market. In this study, all the OCTs have received FDA clearance through the 510(k) pathway.

### Carl Zeiss Cirrus 500, 5000

The Carl Zeiss Cirrus 500 and 5000 (Carl Zeiss Meditec, Inc., Dublin, CA) are Spectral Domain OCT machines with a 5 μm axial resolution and a scan speed of 27,000 to 68,000 scans/second. The Cirrus performs anterior segment, posterior segment, and iris exams [27]. The most recent FDA-approved normative database is for the Cirrus 400 and 4000.

### Normative database

The normative database for the Cirrus includes data for Retinal Nerve Fiber Layer (RNFL), Macula, Optic Nerve Head (ONH), and Ganglion Cell Layer (GCL) [28]. It includes 284 subjects (282 subjects in the macula database) from seven non-specified centers. The demographic distribution of the Cirrus normative database is detailed in Table 1.

**Table 1:** Comparative table of OCT’s normative databases characteristics and demographics

		CIRRUS	AVANTI	SPECTRALIS	TRITON
**Patients, n**		284	480	330	410
**Eyes, n**		284	640	330	410
**Sex (male,) n(%)**		133 (47.2)	215 (44.79)	146 (44.2)	194 (47.3)
**Age, years**	*Range*	18-84	18-84	20-90	18-70+
**Race and ethnicity (%)**	*White*	43	33	79.7	58
	*Black*	18	20	12.42	22
	*Asians*	24	22	7	8
	Hispanic*	12	12	0	0
	*Other*	3	13	0.9	12
**Population country**		Non-specified	Non-specified	Canada, Germany, US	US
**Statistical analysis**		Fitted Regression Model	Pearson Correlation Coefficient Analysis	Multiple Linear Regressions	Quantile Regression

Comparative table of OCT’s normative databases characteristics and demographics

Inclusion criteria were for patients older than 18 years with normal Humphrey Visual Field Test (HVFT) 24 − 2 results and intraocular pressure (IOP) lower than 21 mmHg. Exclusion criteria were patients with best corrected visual acuity (BCVA) worse than 20/40, refractive error outside the ranges of -12.0 to + 8.0, previous ophthalmic laser or incisional surgery, active infection of the anterior or posterior segment, diabetic retinopathy, diabetic macular edema or vitreoretinal disease, diabetes, leukemia, AIDS, systemic hypertension, dementia, or multiple sclerosis.

The statistical strategy applied for the Cirrus normative database was a Fitted Regression Model (expected mean reading (age) + Normative limit (100x %) < Observed reading (age)). The 1st, 5th, and 99th percentiles were estimated by the empirical distribution of residual, and results were grouped and adjusted by age.

### XR RTVue Avanti

The Optovue (Optovue Inc., Fremont, CA) Widefield Avanti is a spectral domain OCT with a 5 μm axial resolution and a scan speed of 70,000 a-scans/second [29]. The Avanti performs anterior segment, posterior segment, and iris exams.

### Normative database

The FDA-approved RTVue Avanti normative databases for ganglion cell complex, RNFL, retinal thickness, optic disc cup, and disc cup were collected from 11 international clinical sites [30, 31]. The dataset comprises 640 eyes of 480 patients aged 18–84 years, and their demographic distribution is detailed in Table 1. The inclusion criteria were participants older than 18 years with a normal HVFT 24 − 2 and IOP less than 22 mmHg. All patients with any ocular pathology were excluded from the normative dataset.

The statistical strategy employed was a Pearson Correlation Coefficient Analysis grouped and adjusted by age, signal strength, and disc area. The RTVue Avanti also has an expanded ethnic database available in the software update to version 4.0, with 861 eyes of patients aged 19–82 years from 15 multinational sites, six in the US, three in China, one in London, three in Japan, and two in India [31]. The reported ethnicity of this expanded database comprised 33% White, 22% Asian, 29% African American, 12% Hispanic, 12% Indian, and 1% other. Similarly, the expanded ethnic normative database area is adjusted by age, signal strength, and disc area, allowing comparison across eight distinct populational groups or a combination of all.

### Heidelberg Spectralis

The Heidelberg Spectralis (Heidelberg Engineering, Inc., Germany) is a Spectral Domain OCT with a 7 μm axial resolution and a scan speed of 40,000 a-scans/second that perform anterior and posterior segment exams.

### Normative database

The Heidelberg Spectralis normative database for RNFL and optic nerve head includes 330 eyes of 330 subjects aged 20–90 years from Canada, Germany, and the US [32]. The demographic distribution is presented in Table 1.

The database inclusion criteria were patients with refractive error between − 6 and + 6 spherical diopters, astigmatism ≤ 2 diopters, IOP ≤ 21 mmHg, and BCVA ≥ 20/40. The exclusion criteria were those with prior intraocular surgery (excluding cataract or LASIK), vitreoretinal disease, diabetic retinopathy, and optic disc disease. The statistical strategy applied was Multiple Linear Regressions method adjusted by age and Bruch’s Membrane Opening area.

### Topcon DRI Triton

The Topcon DRI OCT Triton (DRI-OCT Triton, Topcon Inc., Japan) is a Swept-source OCT with an 8 μm optical and 2.6 μm digital axial resolution and a scan speed of 100,000 a-scans/second.

### Normative database

The DRI Triton normative database for full retinal thickness, RNFL, GCL, GCL plus the inner plexiform layer thickness, and the optic disc, includes 410 patients and eyes ranging from 18 to 70 + years, collected from six clinical sites in the US [33]. The demographic distribution is detailed in Table 1.

The inclusion criteria were patients of more than 18 years with normal eyes, and the exclusion criteria were those with glaucomatous optic nerve damage according to the hemifield visual field test.

The applied statistical strategy employed Quantile Regression with age and/or disc area as regression covariates.

### Diversity

Diversity is an important factor when evaluating OCT’s normative databases. In terms of race, the Avanti dataset has the highest diversity index, followed by Cirrus, Triton, and Spectralis. However, race and ethnic descriptions are not consistently reported in every country, with descriptions of only three of the ten most populous countries (China, USA, and Brazil), which limits the analysis of diversity indexes.

All the OCT datasets have similar sex diversity indexes. This is consistent with the diversity index observed in the World population and in the ten more populated countries (Table 2).

**Table 2:** Comparison of OCT normative databases diversity indexes, world population, and Open-angle glaucoma cohort

Diversity Index		Sex	Race
**OCT**	*Cirrus*	0.692	1.377
	*Avanti*	0.688	1.545
	*Spectralis*	0.687	0.668
	*Triton*	0.692	1.144
**World Population**		0.693	-
	*China*	0.693	0.056
	*India*	0.692	-
	*USA*	0.693	1.185
	*Indonesia*	0.693	-
	*Pakistan*	0.693	-
	*Brazil*	0.693	1.091
	*Nigeria*	0.693	-
	*Bangladesh*	0.693	-
	*Russia*	0.690	-
	*Mexico*	0.693	-
**Glaucoma**		0.688	1.239

Comparison of OCT normative databases diversity indexes, world population, and Open-angle glaucoma cohort

In the open-angle glaucoma meta-analysis diversity index comparison, the sex index is similar across all OCTSs, while the race index is closer to the Triton and Cirrus. The Triton datasets include only patients from the USA, while Cirrus and Avanti datasets do not specify the patient’s nationality. In contrast, the Spectralis dataset includes multinational participants.

Although the Shannon diversity index may not always indicate the generalizability of datasets, it is important to take diversity into account when interpreting OCT results to ensure that diagnostic and treatment decisions are not biased toward a specific group or population.

## Discussion

In this study, we highlight the limitations of fairness and generalizability of Cirrus, Avanti, Spectralis, and Triton normative databases. These limitations must be considered when interpreting OCT’s reports.

All OCTs included in the study provide normative databases for RNFL and optic disc parameters. The Cirrus, Avanti, and Triton machines include macular parameters, and the Cirrus, Avanti, and Triton include GCL comparison.

Diversity is critical for promoting the generalizability of results. Among the OCTs evaluated, the RTVue Avanti OCT has the largest normative database, with data from 640 eyes, and the option to upgrade to an expanded ethnic database is larger still (with 861 eyes), with eight different ethnicity categories. However, the number of included patients is still limited, considering the multiple demographic strata.

Race and ethnicity are not routinely collected in many countries. Nevertheless, to promote fairness and generalizability to minorities, improving demographic data collection is desirable. The applied classification is not uniform across the evaluated normative databases, and following standards is needed. The Cirrus and Avanti databases include Hispanic and Indian as a subdivision of race. The American Standards for classification of Race and Ethnicity define five minimum categories for race (American Indian or Alaska Native, Asian, Black or African American, Native Hawaiian or Other Pacific Islander, and White) and two categories for ethnicity (“Hispanic or Latino” and “Not Hispanic or Latino”) [22]. Perhaps more important to note is the inconsistency in racial/ethnic representation of the normative databases between OCTs, which may differ from populations for whom the normative databases are used.

The Spectralis normative database has a higher proportion of White representation (79.7%), whereas the Cirrus normative database has a higher proportion of Asian patients representation (24%), and the Triton normative database has more Black representation (22%). None of the databases are perfectly representative of the overall background US population, although the Spectralis is the most similar to overall US racial and ethnic demographics from the 2020 census data [34, 35].

Given the demonstrable architectural variations in retinal layers between patients of different races and/or sex, it is crucial to understand whether the patient’s retinal architecture is actually being compared to what is ‘normal’ for someone of that demographic. For example, studies demonstrated that Black women have demonstrably thinner central retinal thickness [36], and African Americans have a significantly lower mean foveal thickness (vs. White/Hispanic), with males having a significantly higher mean foveal thickness (regardless of race) [37–43]. These studies outline the importance of carefully accounting for sex and ethnicity when interpreting OCT scans in the clinical setting and emphasize how consequential a poorly-representative normative dataset.

Pediatric populations (< 18 years old) were not included in any normative database, making the assessment of severity/deviation from ‘normal’ difficult in pediatric populations and emphasizing the need for normative pediatric databases [21, 44]. Elderly populations, which are the focus of studies evaluating dementia and Alzheimer’s disease, also are underrepresented in OCT normative databases [45, 46]. Leading to problematic generalizability in these groups.

While all normative databases apply traditional regression models stratified by age and/or optic disc size, none stratify according to ethnicity or sex, and more nuanced modeling techniques (i.e., machine learning algorithms) might help overcome biases posed by poorly-representative datasets were not applied in any regression analyses.

The Shannon diversity measurement evaluates the entropy among groups, however, do not reflect the fairness or composition among the demographic distribution. Although the USA demographic is closer to the Spectralis normative database, the Triton diversity index is closer to the diversity of races distribution within Brazil and the USA.

Collaborative international research using publicly-available datasets in maximizing the safety and utility of Artificial Intelligence algorithms to healthcare has been described in detail elsewhere [47]. Publicly available data represents a possible means for overcoming pitfalls posed by limited sociodemographic representation in OCT datasets and facilitates collaborative research and validation studies. However, none of the manufacturer’s normative database data are publicly available among the OCT machines described herein,

As the scope for the use of technology in healthcare grows, an appreciation for the risks of unfair results is crucial. In order to optimize the accuracy of OCT interpretation, representative normative datasets and adjustments for patients’ unique sociodemographic qualities are essential. In the meantime, sharing datasets and international collaboration can help illuminate how technology can be sensitively applied to marginalized populations and may help to mitigate the risk of these tools propagating current healthcare disparities.

Our study has some limitations. Firstly, our analysis relies on publicly available data from OCT manufacturers, which does include detailed information about demographics and measurement values, limiting the comparison. Secondly, although all machines use similar principles of physics, what the machines deem ‘normal,’ how results are shown, and retinal and optic nerve head boundaries vary widely between machines and therefore are not interchangeable [37, 48]. Lastly, the diversity index only evaluates the entropy among groups and does not reflect the fairness or composition of the demographic distribution.

In conclusion, using digital ancillary imaging exams is becoming widespread in healthcare, with OCT helping diagnose and manage many ophthalmic diseases. The in-built normative databases in OCT equipment have limitations in fairness and generalizability when they are not representative of the patient populations for whom they are used. As a result, caution in OCT normality interpretation is warranted. To address these limitations, there is a need for more diverse, representative, and open-access datasets that take into account patient demographics, especially considering the development of supervised Machine Learning algorithms in healthcare.

## Data Availability

all data that support this study’s findings are publicly available at www.accessdata.fda.gov.
